# Validation of the PROMIS Sleep Disturbance and Sleep-Related Impairment item banks in Dutch adolescents

**DOI:** 10.1007/s11136-018-1856-x

**Published:** 2018-04-16

**Authors:** Jojanneke A. M. C. van Kooten, Raphaёle R. L. van Litsenburg, Whitney R. Yoder, Gertjan J. L. Kaspers, Caroline B. Terwee

**Affiliations:** 1 Department of Pediatric Oncology - Hematology, VU University Medical Centre Amsterdam, Amsterdam, The Netherlands; 2 Department of Epidemiology and Biostatistics and Amsterdam Public Health Research Institute, VU University Amsterdam, P.O. box 7057, 1007 MB Amsterdam, The Netherlands; 3 Princess Máxima Center for Pediatric Oncology, Utrecht, The Netherlands

**Keywords:** Sleep, Adolescent, Child, PROMIS, Validation

## Abstract

**Purpose:**

Sleep problems are common in adolescents and have a negative impact on daytime functioning. However, there is a lack of well-validated adolescent sleep questionnaires. The Patient-Reported Outcomes Measurement Information System (PROMIS) Sleep Disturbance and Sleep-Related Impairment item banks are well-validated instruments developed for and tested in adults. The aim of this study was to evaluate their structural validity in adolescents.

**Methods:**

Test and retest data were collected for the Dutch–Flemish V1.0 PROMIS Sleep Disturbance (27) and Sleep-Related Impairment (16 items) item banks from 1046 adolescents (11–19 years). Cross-validation methods, Confirmatory (CFA), and Exploratory Factor Analyses (EFA) were used. Fit indices and factor loadings were used to improve the models. The final models were assessed for model fit using retest data.

**Results:**

The one-factor Sleep Disturbance (CFI = 0.795, TLI = 0.778, RMSEA = 0.117) and Sleep-Related Impairment (CFI = 0.897, TLI = 0.882, RMSEA = 0.156) models could not be replicated in adolescents. Cross-validation resulted in a final Sleep Disturbance model of 23 and a Sleep-Related Impairment model of 11 items. Retest data CFA showed adequate fit for the Sleep-Related Impairment-11 (CFI = 0.981, TLI = 0.976, RMSEA = 0.116). The Sleep Disturbance-23 model fit indices stayed below the recommended values (CFI = 0.895, TLI = 0.885, RMSEA = 0.105).

**Conclusions:**

While the PROMIS Sleep Disturbance-23 for adolescents and PROMIS Sleep-Related Impairment-11 for adolescents provide a framework to assess adolescent sleep, additional research is needed to replicate these findings in a larger and more diverse sample.

## Introduction

Due to biological and social differences in adolescents, sleep patterns tend to be different than those of young children and adults [1–3]: both sleep duration and sleep quality are considered frequently insufficient in this population. Sleep problems are reported by 6–23% of adolescents [4–6]. The large range of self-reported sleeping problems is due to varying definitions of “sleep problems” between studies, based on different questionnaires or single questions. According to the 2016 guideline of the American Academy of Sleep Medicine, young adolescents (aged 11–12) require 9–12 h of sleep per night and older adolescents (13–18) require 8–10 h [7]. However, more than half of adolescents sleep less than recommended [8, 9]. Moore et al. consider this the result of the interaction between puberty and academic, social, and extracurricular demands [10]. These results are confirmed by other studies [2, 8, 11]. Arora et al. subsequently added technology to the explanatory factors [12]. In otherwise healthy adolescents, insufficient sleep and sleepiness significantly impact daytime functioning and therefore critical functions in adolescent development such as academic performance, memory performance, mood, and risk-taking behaviors [8, 10, 13, 14]. In children suffering from chronic illnesses such as cancer, sleep problems are common clinical symptoms and are known to negatively influence quality of life [15–17]. Therefore, identification and monitoring of sleep problems and the consequences of sleep problems are important for both the healthy and sick adolescent population.

Questionnaires are suitable to measure perceived sleep problems and consequences of sleep problems, such as fatigue, daytime sleepiness, and bedtime resistance [18]. The many factors known to influence adolescent sleep make the need for questionnaires with carefully selected content and good psychometric properties. A recent systematic review by Ji et al. identified 13 instruments used to evaluate sleep quality and Sleep Disturbances in adolescents between 2000 and 2016. Although most of the instruments have been evaluated for reliability, none of these questionnaires had evidence for all essential measurement properties. The authors therefore could not recommend any of the questionnaires for future use [19].

The Patient-Reported Outcomes Measurement Information System (PROMIS) sleep item banks can be considered psychometrically promising measures and therefore a possible alternative for the existing questionnaires [20, 21]. PROMIS is a large initiative that has developed an innovative assessment system devoted to measuring patient-reported health [22–24]. PROMIS consists of a collection of item banks, which are large sets of items measuring one aspect of health status, such as physical functioning, anxiety, or sleep problems. Item banks are developed using modern psychometric methods, called Item Response Theory methods. Items can be selected from an item bank to be used as short forms, but item banks also offer the advantage of computerized adaptive testing (CAT), in which an assessment can be tailored to the individual person [25, 26]. A CAT is a computer-administered test in which, after the first item, presentation of items is determined by persons’ responses to previous ones. This smart and efficient measurement allows patients to only respond to a minimal number of relevant items.

Included in the PROMIS system are two item banks that aim to assess Sleep Disturbance and Sleep-Related Impairment [20, 21]. These item banks have been developed for adults and have shown good psychometric properties [20]. A recent study by Hanish et al. aimed to assess the clinical utility of both the Sleep Disturbance and Sleep-Related Impairment 8-item short forms in 25 adolescents [27]. They deemed further research necessary to determine the value in adolescents. In view of the PROMIS sleep item banks’ robustness in adults and international implementation of PROMIS in practice and research [28–32], it is important to fully assess the usefulness of these same item banks in an adolescent population. Providing a valid and efficient assessment tool for measuring sleep problems and their consequences in adolescents would advance the field and provide health care communities with a much needed method of recognizing sleep problems in adolescents.

Primary validation steps for the use of these item banks in adolescents were previously taken. Van Kooten et al. reported on the content validity by assessing relevance, comprehensiveness, and comprehensibility of the items of self- and proxy versions of the PROMIS Sleep Disturbance and PROMIS Sleep-Related Impairment item banks in Dutch adolescents. It was concluded that both item banks are useful in assessing adolescent sleep but psychometric evaluation of both scales is needed [33]. Therefore, the aim of this study is to evaluate the structural validity of the Dutch–Flemish PROMIS V1.0 Sleep Disturbance and V1.0 Sleep-Related Impairment item banks in adolescents.

## Methods

### Study participants

Adolescents were recruited from secondary schools (age range 11–19 years) in the Netherlands, between February 2014 and March 2015. Schools from all educational levels and from different regions of the Netherlands were included. We aimed to include 1000 students as recommended by PROMIS for calibration analyses [34].

### Study procedures

Adolescents were asked to fill out the Dutch–Flemish PROMIS Sleep Disturbance item bank and Sleep-Related Impairment item bank during regular class hours. In addition, descriptive data were collected on gender, age, and education level. The study questionnaires were filled out online. In one school online entry was not possible due to lack of digital resources, so paper versions were distributed here (*n* = 164). There were no differences in missing answers per item and *T* scores were comparable between both groups. During online and paper administration, author JvK was present in the classroom to supervise the procedure.

To enable cross-validation, all participants were invited to fill out the questionnaire for a second time. Participants that were interested could do so by filling out their e-mail address at the end of the questionnaire. Two weeks after the first assessment (for both paper and online participants), a link to the repeat questionnaire was sent via e-mail.

### Item banks

The PROMIS Sleep Disturbance and Sleep-Related Impairment item banks were developed based on literature reviews, content-expert advice, focus groups with 36 people with and without sleep problems to ensure relevance and comprehensiveness, and cognitive interviews with 20 participants to examine comprehensibility [20]. The PROMIS Sleep Disturbance item bank contains 27 items that are reflective of insomnia-like items and assesses one’s perception of their sleep quality and restoration associated with sleep, perceived sleep difficulties and concerns with falling and staying asleep, and perceptions of adequate and satisfactory sleep. The PROMIS Sleep-Related Impairment item bank consists of 16 items that are related to patient sleepiness, fatigue, and cognitive difficulties during waking hours. In addition, Sleep-Related Impairment items assess perceptions of functional impairment during waking hours that are associated with sleep problems or impaired alertness. It is important to note that these item banks do not measure symptoms of specific sleep disorders as the intent is to rather gain a more general overview of one’s perception of sleep problems and how these problems hinder daily functioning [20, 35].

Traditional psychometric methods (such as factor analysis) and IRT analyses have been used to assess their psychometric properties in a sample of 1993 adults from the general American population (including 734 people with self-reported sleep problems and 1259 without sleep problems) and a clinical sample of 259 patients with sleep problems [20].

All Sleep Disturbance and Sleep-Related Impairment items are measured on a 5-point Likert scale (1 = Not at all or Never, 2 = A little bit or Rarely, 3 = Somewhat or Sometimes, 4 = Quite a bit or Often, 5 = Very much or Always) and are indicative of how frequently respondents have experienced problems related to sleep in the last 7 days.

This study used the Dutch–Flemish versions V1.0 of the item banks [29], of which the content validity was found to be acceptable for use in adolescents [33]. Studying the content validity, we identified two items in the Sleep Disturbance item bank (items Sleep116 *“My sleep was refreshing”* and Sleep87 *“I had trouble staying asleep”*) which were difficult to understand by adolescents. These items were rephrased (now called Sleep116a “*My sleep gave me renewed energy*” and Sleep87a “*I had trouble sleeping through the night*”) [33]. The rephrased items were used in the current analyses.

### Statistical analyses

Adolescents who did not complete the full item bank were excluded from the analyses because the software used (Mplus version 7) cannot handle missing values. For seven Sleep Disturbance items and three Sleep-Related Impairment items, the response categories were reversed as to make sure that higher scores indicate more of the construct measured. Response category frequencies were examined per item and response categories were collapsed for any items that had a response category with 5% or less responses. An initial confirmatory factor analysis (CFA) was conducted in Mplus on the polychoric correlation matrix with weighted least squares with mean and variance adjustment (WLSMV) estimation for each item bank separately, to examine the expected unidimensionality of the item banks. For evaluating model fit, the Comparative Fit Index (CFI), Tucker–Lewis Index (TLI), and Root Means Square error or Approximation (RMSEA) were used.

When model fit for an item bank was not found to meet the recommended criteria of CFI > 0.95, TLI > 0.95, and RMSEA < 0.06, [36] an exploratory approach similar to what was used in the item banks’ primary development procedure was adopted [20]. This provided the opportunity to further assess the dimensionality and to understand the misfit of the model in adolescents. A cross-validation procedure was used for this purpose. First, each data set was randomly split into two subsamples. The first subsample was used to conduct an exploratory factor analysis (EFA) and the second for a subsequent CFA. EFAs were conducted using Mplus with Promax Rotation and WLSMV estimation procedures. These choices were reflective of the choices made in the initial validation study [20]. A unidimensional factor model was assumed sufficient when the first factor accounted for at least 20% of the variability and when the ratio of the variance explained by the first to the second factor was greater than four [36, 37]. Scree plots and Eigenvalues were studied to examine if more than one factor should be considered. Response distributions and factor loadings were studied to examine if the model could be improved by further collapsing response options or removing misfitting items from the model. Item content was also discussed to find an explanation for misfitting items.

Once decisions were made to improve the model fit by removing misfitting items, a CFA of the new model was conducted on the second subsample. Model fit parameters (CFI, TLI, RMSEA) and item factor loadings were evaluated to examine if further improvement of the model was possible. In the last step, the final model that was extracted from the cross-validation procedures for both PROMIS sleep item banks was validated in a CFA conducted on retest data.

## Results

### Sample characteristics

In total, 1046 adolescents from seven different schools participated in the study. Complete data were available for 947 (91%) adolescents on the Sleep Disturbance item bank and 958 (92%) on the Sleep-Related Impairment item bank. Of the 1046 included adolescents, 372 (35.6%) filled out their e-mail address and a total of 124 (11.9%) eventually completed the PROMIS item banks again after 2 weeks. 108 participants (87%) responded to the complete set of items.

Sample characteristics are summarized in Table 1. Adolescents in the test and retest sample ranged in age from 11 to 19 with a mean age (SD) of 14 years (1.5) in the test sample. The age distribution in the test sample seems equal to the general population according to Statistics Netherlands: Our population contains 41.3% young students in the bridge year, versus 42.4% in the general population. Approximately 48% of respondents were boys (*n* = 503) in the test sample (33% in the retest sample). The percentage of boys in the general population is 50% [38].

**Table 1:** Demographic characteristics of Dutch adolescent samples

	Test sample	Retest sample
Number	*n* = 1046	*n* = 124
Missing		
Sleep Disturbance	*n* = 99 (9%)	*n* = 16 (13%)
Sleep-Related Impairment	*n* = 88 (8%)	*n* = 16 (13%)
Mean age (SD)	14 (1.53)	14 (1.48)
Age range	11–19	11–19
Male (%)	503 (48.1%)	42 (33.9%)
Educational level^a^		
Bridge year	432 (41.3%)	33 (26.6%)
Lower level (VMBO)	79 (7.6%)	4 (3.2%)
Middle level (HAVO)	272 (26%)	23 (18.5%)
Higher level (VWO)	255 (24.4%)	63 (50.8%)
Unknown	8 (0.8%)	1 (0.8%)
Mean *T* score		
Sleep Disturbance	50.27 (9.13)	50.41 (9.41)
Sleep-Related Impairment	50.22 (9.10)	50.46 (9.17)

*HAVO* Hoger Algemeen Voortgezet Onderwijs (middle level of Dutch secondary school), *VMBO* Voorbereidend Middelbaar Beroeps Onderwijs (lowest level), *VWO* Voorbereidend Wetenschappelijk Onderwijs (highest level)

^a^Educational levels are based on the differentiation as made by the Statistics Netherlands. Distribution in the Dutch general population: Bridge year (year 1–2/1–3 students who have not made a choice yet as to which level they will pursuit eventually) 42.4%; VMBO 21.5%; HAVO 16.0%; VWO 17.2% [40]

In relation to the Dutch population, our study sample has a higher percentage of adolescents with the middle and highest level of education (Table 1). Adolescents with the highest level of education also tended to respond more often to the retest: 24% in the test sample and 50% in the retest sample had the highest level of education [38].

### Initial confirmatory factor analysis

An initial CFA was conducted on both sleep item banks (Tables 2, 3). Since the sample was extracted from a general population, responses were skewed, with most adolescents reporting low levels of sleep problems. For the Sleep Disturbance item bank, 21 out of 27 items were therefore collapsed to four categories (1 = Not at all or Never, 2 = A little bit or Rarely, 3 = Somewhat or Sometimes, 4 = Quite a bit/Very much, Often/Always) and two other items (Sleep68 and Sleep70) were collapsed to three categories (1 = Not at all or Never, 2 = A little bit or Rarely, 3 = Somewhat/Quite a bit/Very much or Sometimes/Often/Always). Only items Sleep115, Sleep42, and Sleep69 were not collapsed. For the Sleep-Related Impairment item bank, 10 out of 16 items (Sleep7, Sleep10, Sleep11, Sleep18, Sleep19, Sleep25, Sleep27, Sleep29, Sleep30 and Sleep33) were collapsed to four categories (1 = Not at all or Never, 2 = A little bit or Rarely, 3 = Somewhat or Sometimes, 4 = Quite a bit/Very much, Often/Always). Model fit measures for both PROMIS Sleep Disturbance (CFI = 0.795, TLI = 0.778, RMSEA = 0.117) and PROMIS Sleep-Related Impairment (CFI = 0.897, TLI = 0.882, RMSEA = 0.156) item banks did not confirm the unidimensionality of the item banks.

**Table 2:** Results (model fit parameters and factor loadings) of the exploratory factor analysis (EFA) and confirmatory factor analyses (CFA) of the PROMIS Sleep Disturbance item bank

Item	Content	Initial CFA	EFA		CFA	CFA
		Total sample *N* = 1049	First random subsample *N* = 473		Second random subsample *N* = 473	Retest sample *N* = 124
		Factor 1	Factor 1	Factor 2	Factor 1	Factor 1
CFI		0.795			0.821	0.895
TLI		0.778			0.803	0.885
RMSEA		0.117			0.119	0.105
Sleep105	My sleep was restful…	0.658	0.816	− 0.061	0.671	0.770
Sleep106	My sleep was light…	0.343	**0.272**	**0.174**		
Sleep107	My sleep was deep…	0.375	0.468	0.060	0.290	0.429
Sleep108	My sleep was restless…	0.610	**0.356**	**0.379**		
Sleep109	My sleep quality was…	0.726	0.782	0.052	0.723	0.838
Sleep110	I got enough sleep…	0.603	0.727	0.022	0.552	0.489
Sleep115	I was satisfied with my sleep…	0.705	0.950	− 0.098	0.659	0.701
Sleep116	My sleep was refreshing…	0.468	0.563	0.020	0.438	0.607
Sleep125	I felt lousy when I woke up…	0.511	**0.375**	**0.220**		
Sleep20	I had a problem with my sleep…	0.722	**0.461**	**0.414**		
Sleep42	It was easy for me to fall asleep…	0.750	0.166	0.623	0.781	0.911
Sleep44	I had difficulty falling asleep…	0.825	0.055	0.829	0.823	0.914
Sleep45	I laid in bed for hours waiting to fall asleep…	0.781	0.102	0.726	0.785	0.798
Sleep50	I woke up too early and could not fall back asleep…	0.547	− 0.117	0.664	0.572	0.608
Sleep65	I felt physically tense at bedtime…	0.458	− 0.099	0.530	0.497	0.600
Sleep67	I worried about not being able to fall asleep…	0.721	− 0.027	0.803	0.716	0.655
Sleep68	I felt worried at bedtime…	0.580	− 0.065	0.670	0.577	0.559
Sleep69	I had trouble stopping my thoughts at bedtime…	0.602	− 0.077	0.670	0.641	0.628
Sleep70	I felt sad at bedtime…	0.619	0.022	0.591	0.678	0.575
Sleep71	I had trouble getting in a comfortable position to sleep…	0.592	0.117	0.530	0.593	0.655
Sleep72	I tried hard to get to sleep…	0.652	0.036	0.679	0.638	0.617
Sleep78	Stress disturbed my sleep…	0.657	0.123	0.625	0.640	0.590
Sleep86	I tossed and turned at night…	0.618	0.037	0.619	0.424	0.714
Sleep87	I had trouble staying asleep…	0.645	0.179	0.574	0.601	0.772
Sleep90	I had trouble sleeping…	0.814	0.245	0.641	0.833	0.902
Sleep92	I woke up and had I trouble falling back to sleep…	0.636	− 0.100	0.753	0.650	0.765
Sleep93	I was afraid that I would not get back to sleep after waking up…	0.614	− 0.056	0.719	0.602	0.631

Bold items loaded higher on the second factor than on the first factor in EFA

*CFI* Comparative Fit Index, *TLI* Tucker–Lewis Index, *RMSEA* Root Means Square error or Approximation

**Table 3:** Results (model fit parameters and factor loadings) of the exploratory factor analysis (EFA) and confirmatory factor analyses (CFA) of the PROMIS Sleep-Related Impairment item bank

Item	Content	Initial CFA	EFA		CFA	CFA
		Total sample *n* = 1049	First random subsample *N* = 479		Second random subsample *N* = 479	Retest sample *N* = 124
		Factor 1	Factor 1	Factor 2	Factor 1	Factor 1
CFI		0.897			0.955	0.981
TLI		0.882			0.943	0.976
RSMEA		0.156			0.144	0.116
Sleep4	I had enough energy…	0.525	0.249	**0.365**		
Sleep6	I was sleepy during the daytime	0.798	0.536	0.369	0.825	0.764
Sleep7	I had trouble staying awake during the day…	0.843	0.624	0.355	0.845	0.903
Sleep10	I had a hard time getting things down because I was sleepy	0.842	0.756	0.168	0.838	0.909
Sleep11	I had a hard time concentrating because I was sleepy	0.878	0.828	0.116	0.885	0.904
Sleep18	I felt tired…	0.830	0.628	0.322	0.817	0.797
Sleep19	I tried to sleep whenever I could…	0.555	0.562	0.080	0.531	0.694
Sleep25	I had problems during the day because of poor sleep…	0.855	0.896	− 0.006	0.855	0.931
Sleep27	I had a hard time concentrating because of poor sleep	0.902	0.864	0.075	0.911	0.913
Sleep29	My daytime activities were disturbed by poor sleep	0.832	0.873	0.033	0.799	0.863
Sleep30	I felt irritable because of poor sleep…	0.705	0.820	− 0.100	0.699	0.873
Sleep33	I had a hard time controlling my emotions because of poor sleep…	0.718	0.877	− 0.161	0.734	0.851
Sleep119	I felt alert when I woke up…	0.337	− 0.096	**0.509**		
Sleep120	When I woke up I felt ready to start the day…	0.606	0.061	**0.651**		
Sleep123	I had difficulty waking up…	0.623	− 0.096	**0.812**		
Sleep124	I still felt tired sleepy when I woke up	0.690	0.021	**0.801**		

Bold items loaded higher on the second factor than on the first factor in EFA

*CFI* Comparative Fit Index, *TLI* Tucker–Lewis Index, *RMSEA* Root Means Square error or Approximation

### Cross-validation

Data sets for both item banks were randomly split into two subsamples. A random split of the PROMIS Sleep Disturbance and PROMIS Sleep-Related Impairment data sets resulted in two equal subsamples of 473 (Sleep Disturbance) and 479 (Sleep-Related Impairment) adolescents.

### Sleep Disturbance

**Fig. 1:**
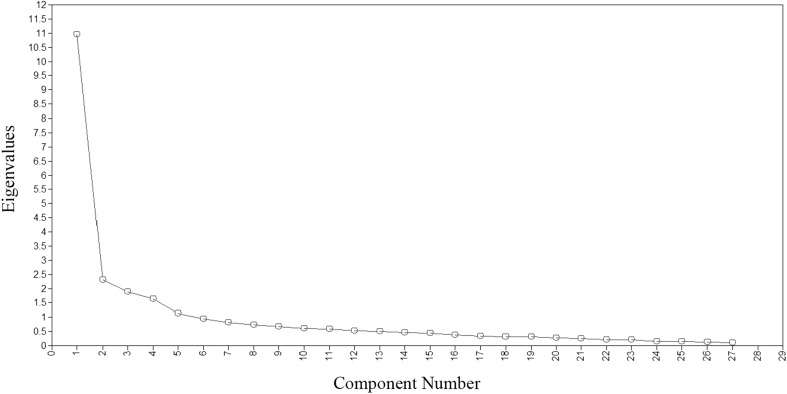
Scree plot extracted from Sleep Disturbance exploratory factor analysis

One additional item (Sleep44) was collapsed to four categories, and one item (Sleep87a) was further collapsed to three categories. Initial EFA on the first random subsample showed a second elbow at four factors in the scree plot (Fig. 1 Scree plots extracted from Sleep Disturbance exploratory factor analysis), suggesting problems with unidimensionality. A two-factor model was examined since two factors (Sleep Onset and Sleep Quality) were combined in the original validation article [20]. The eigenvalues of the first two factors were 10.96 and 2.30, respectively, and the ratio of the first to the second factor was 4.77. Three items that originally measured sleep onset (Sleep106: “My sleep was light,” Sleep108: “My sleep was restless,” Sleep125: “I felt lousy when I woke up”) and one item related to quality of sleep (Sleep20: “I had a problem with my sleep”) were flagged as problem items because they loaded on both factors with low factor loadings (Table 2). Further, Sleep20 loaded slightly higher on the sleep onset factor when it originally was found to be an indicator of sleep quality. This suggests that these items do not appropriately measure the original two Sleep Disturbance constructs in adolescents or they in fact are measuring another latent trait. Therefore, the two-factor model was conceptually rejected. The item bank was further explored for three or four factors. However, the EFA results did not clearly indicate any other meaningful factors. Because it was not possible to find a meaningful multi-dimensional factor structure, it was decided to perform a one-factor CFA on the second random subsample without the four problematic items mentioned above. The one-factor model on the Sleep Disturbance-23 showed better model fit (CFI = 0.821, TLI = 0.803, RMSEA = 0.119), though fit indices were well outside the recommended range. The factor loading of item Sleep107: “My sleep was deep” was low (0.290). This item was not (yet) deleted however, because we would like to examine its performance in a new sample before making this decision.

### Sleep-Related Impairment

**Fig. 2:**
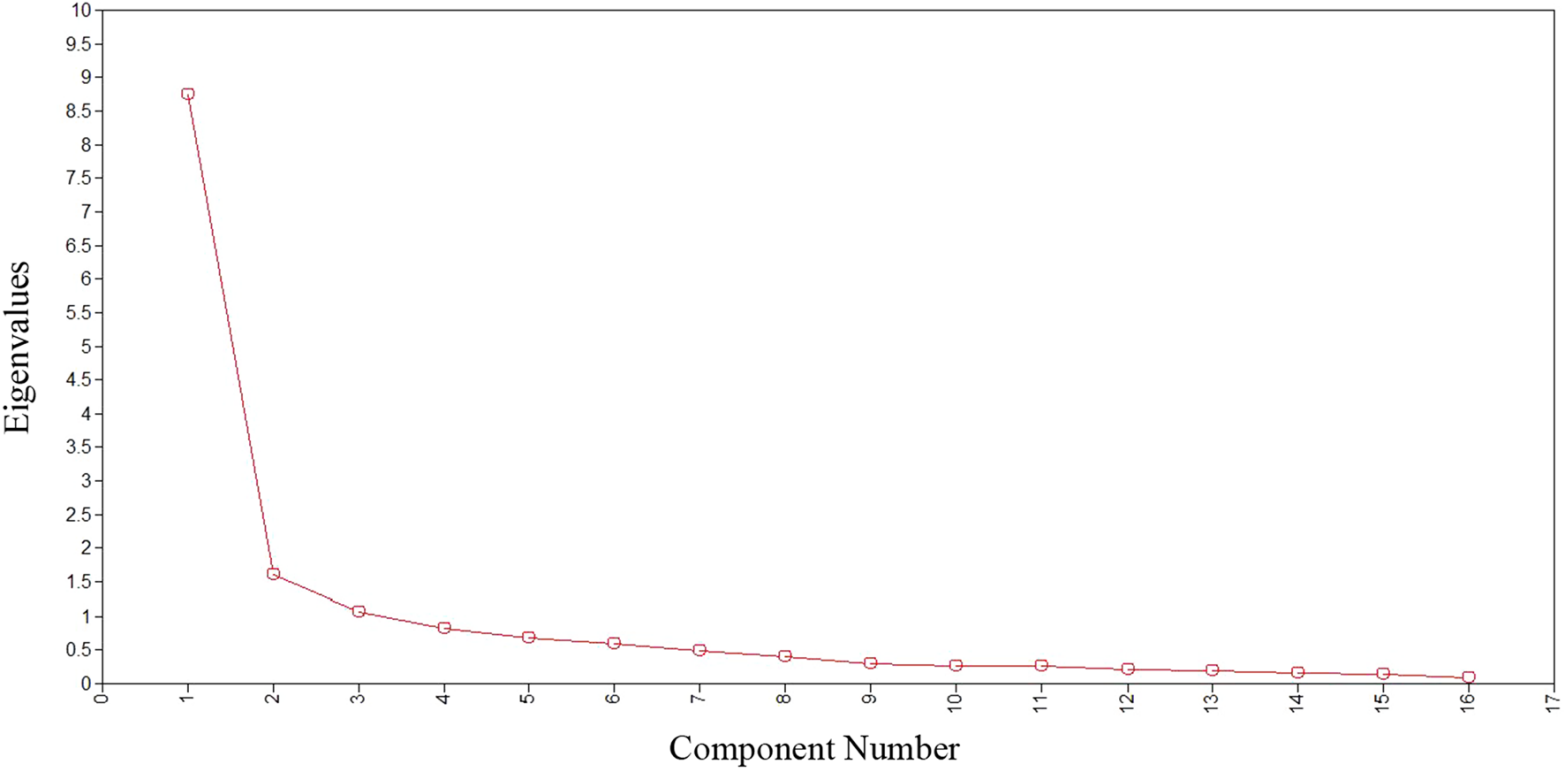
Scree plot extracted from Sleep-Related Impairment exploratory factor analysis

One additional item (Sleep6) was collapsed to four categories, and two items (Sleep25 and Sleep29) were further collapsed to three categories. Initial EFA on the first random subsample showed one dominant factor and an elbow after two factors for the Sleep-Related Impairment items in the scree plot (Fig. 2 Scree plots extracted from Sleep-Related Impairment exploratory factor analysis). As two factors (Waking Symptoms and Sleep-Wake Transition) were originally combined during primary construction of the Sleep-Related Impairment item bank [20], it was sought to assess the use of a two-factor model. The eigenvalues of the first two factors were 8.74 and 1.61, respectively, and the ratio of the first to the second factor was 5.43. In EFA, item factor loadings matched those of the two factors described in the original validation article. Five items loaded on a second Waking Symptoms factor (Table 3). An explanation could lay in three of these five items being reversely scored (Sleep4, Sleep119, Sleep120). Another explanation might be that waking up for adolescents is known to be difficult in the first place, e.g., circadian rhythm disruptions and delayed sleep phase disorder are common [2, 3, 39]. Since these five items were considered conceptually problematic and not clearly measuring a different construct, we consider this second factor not meaningful. Therefore, it was decided to perform a one-factor CFA on the second random subsample without these five items. The one-factor model on the Sleep-Related Impairment-11 showed better model fit (CFI = 0.955, TLI = 0.943, RMSEA = 0.144). Upon examination of factor loadings, item Sleep19: “I tried to sleep whenever I could” loaded quite low (0.531) on the dominant factor compared to the other items (all 0.80 or higher). As sample size was small due to the sample split and the factor loading was just above 0.5, it was decided to keep this item.

### Confirmatory factor analysis in retest data

The final PROMIS Sleep Disturbance-23 for adolescents and PROMIS Sleep-Related Impairment-11 for adolescents item banks were assessed for model fit using data collected from the same sample 2 weeks after primary data collection. For the CFA of the Sleep Disturbance-23, one more item (Sleep42) needed to be collapsed to four categories.

CFA of the Sleep-Related Impairment-11 indicated good model fit with fit indices of adequate size (CFI = 0.981, TLI = 0.976, RMSEA = 0.116) (Table 3). Although the RMSEA value was outside of recommended criteria, it can be said that the final Sleep-Related Impairment-11 proves to be adequate indicators of adolescent sleep. CFA of the Sleep Disturbance-23 indicated decent model fit (CFI = 0.895, TLI = 0.885, RMSEA = 0.105) (Table 2), but fit indices were still well below the recommended values. Flagged in the previous CFA analyses, Sleep Disturbance item Sleep107 and Sleep-Related Impairment item Sleep19 seemed to perform quite well in this CFA analyses but the factor loading of Sleep-Related Impairment item Sleep19 was still quite low when compared to the other Sleep-Related Impairment item factor loadings (0.429). These results suggest the need for further evaluation of item content and use in this specific population.

## Discussion

The unidimensionality of the PROMIS V1.0 Sleep Disturbance and V1.0 Sleep-Related Impairment item banks could not be replicated in Dutch adolescents. Cross-validation resulted in two final item banks: “PROMIS V1.0 Sleep Disturbance-23 for adolescents” and “PROMIS V1.0 Sleep-Related Impairment-11 for adolescents” with decent model fit (CFI = 0.895, TLI = 0.885, RMSEA = 0.105) and good model fit (CFI = 0.981, TLI = 0.976, RMSEA = 0.116), respectively. The item banks should be considered preliminary because IRT analyses have not yet been performed.

It should be noted that model fit indices for earlier versions of the item banks were also slightly outside of the desired range. Buysse et al. reported a CFI of 0.843, TLI of 0.957, and RMSEA of 0.140 for a preliminary set of 39 Sleep Disturbance items in adults. A CFI of 0.812, TLI of 0.955, and RMSEA of 0.157 were reported for a preliminary set of 33 Sleep-Related Impairment items in adults [20]. This suggests that the item banks may not measure one construct, although misfitting items may have been removed from the final item banks. Fit indices have not been reported for the final V1.0 Sleep Disturbance and V1.0 Sleep-Related Impairment item banks.

Cross-validation procedures were useful in identifying a set of five Sleep-Related Impairment items that were related to problems with waking up, to which respondents answered differently than the other Sleep-Related Impairment items. Between 30 and 54% of the adolescents reported quite a bit or very much problems with waking up. Research has shown that adolescents often do not get a sufficient amount of sleep and therefore experience problems with waking up [9, 10, 39, 40]. Therefore, these items may not discriminate between adolescents with and without sleep problems. Removing these items resulted in a better model fit in the cross-validation analyses, as well as in the retest data.

Further research is needed to fully comprehend the performance of the PROMIS Sleep Disturbance and Sleep-Related Impairment item banks in adolescents with and without known sleep problems. Of particular focus should be the items that were deleted from the item banks, confirming that they inadequately measure sleep problems for this age group. Items that performed poorly in the final CFA should also be reviewed statistically and conceptually and revised or removed if necessary. Although item content for both translated item banks were considered relevant, comprehensive, and comprehensible [33], it would also be beneficial to confirm that differences are due to age and not due to culture or translation. Research into cultural differences in adolescent sleep patterns has been mostly focused on sleep time: Asian adolescents sleep significantly less than Americans, who in turn sleep significantly less than European and Australian adolescents. Cultural practices, such as the competitive educational system in most Asian countries and the earlier school start times in USA, are thought to influence these bed times. Research into the consequences of inadequate sleep has been strongly limited by a large variety in methods to asses these consequences. The available data suggest tendency for higher rates of daytime sleepiness in Asian adolescents [1, 41]. To evaluate if differences are due to age or cultural aspects, it is important to also validate the Dutch–Flemish Sleep Disturbance and Sleep-Related Impairment item bank in an adult Dutch general population, and to test for differential item functioning (DIF) between adolescents and adults. IRT properties of finalized questionnaires must also be studied and reported in order to support the use of CAT versions in the future. Researchers from the Children’s Hospital of Philadelphia have recently developed PROMIS Pediatric Sleep Health Item Banks for children ages 8 and up [42]. Future studies should investigate what the most suitable instruments are for adolescents.

This study is limited by a general adolescent population. Without including adolescents with known sleep problems, it was not possible to assess the performance of the highest categories of the items. This would have brought us a further understanding of their ability to effectively measure sleep problems in adolescents with and without sleep problems. The current sample is representative of the general adolescent population in the Netherlands regarding age and sex distribution. All educational levels are represented, though our sample has a higher percentage of higher level education students.

Second, the performance of the final models was assessed in a relatively small retest dataset. Previous research has shown that response rates in surveys are generally low and have been declining. Sax et al. reported a 17–24% response rate in web- and paper-based surveys in a group of over 4000 freshman college students [43]. In our sample, only 372 out of 1046 students provided their e-mail address and 124 of them completed the retest, resulting in a final response rate of 33%. We do not have information on the specific reason for participants not to provide an e-mail address or failing to fill out the retest. However, a reason could be that students were asked to fill out the retest at home, as it was unfortunately not possible to also fill out the retest during school hours. Third, IRT analyses were not yet performed due to uncertainty regarding unidimensionality of the item banks, and thus further research will be necessary.

In summary accurately measuring sleep problems in adolescents is needed, but requires the use of validated instruments. The one-factor models for the PROMIS V1.0 Sleep Disturbance and V1.0 Sleep-Related Impairment item banks could not be replicated in a Dutch adolescent population. With the use of cross-validation procedures, revisions were made to improve the Sleep-Related Impairment item bank, resulting in a useful model of 11 items. However, it was difficult to find a model of adequate model fit for the Sleep Disturbance item bank. Although the final PROMIS Sleep Disturbance-23 for adolescents and PROMIS Sleep-Related Impairment-11 for adolescents item banks provide the groundwork in which clinicians can begin assessing adolescent sleep, additional research is needed to identify optimal instruments for assessing Sleep Disturbance and Sleep-Related Impairment in adolescents.
